# Activation of AMPK by* Buddleja officinalis* Maxim. Flower Extract Contributes to Protecting Hepatocytes from Oxidative Stress

**DOI:** 10.1155/2017/9253462

**Published:** 2017-04-03

**Authors:** Ji Yun Jung, Chul Won Lee, Sang Mi Park, Kyung Hwan Jegal, Jae Kwang Kim, Chung A. Park, Il Je Cho, Dae Hwa Jung, Won G. An, Sae Kwang Ku, Rongjie Zhao, Sang Chan Kim

**Affiliations:** ^1^MRC-GHF, College of Korean Medicine, Daegu Haany University, Gyeongsan 38610, Republic of Korea; ^2^HaniBio Co., Ltd., Gyeongsan 712-260, Republic of Korea; ^3^Division of Pharmacology, School of Korean Medicine, Pusan National University, Yangsan 626-870, Republic of Korea; ^4^School of Mental Health, Qiqihar Medical University, Qiqihar, Heilongjiang 161042, China

## Abstract

The* Buddleja officinalis *Maxim. flower is used in traditional Chinese and Korean medicine to treat inflammation, vascular diseases, headache, and stroke, as well as enhance liver function. This research investigated the effects of* B. officinalis* Maxim. flower extract (BFE) on hepatotoxicity. The cytoprotective effects and mechanism of BFE against severe mitochondrial dysfunction and H_2_O_2_ production in hepatotoxicity induced by coadministration of arachidonic acid (AA) and iron were observed in the HepG2 cell line. In addition, we performed blood biochemical, histopathological, and histomorphometric analyses of mice with carbon tetrachloride- (CCl_4_-) induced acute liver damage. BFE inhibited the AA + iron-mediated hepatotoxicity of HepG2 cells. Moreover, it inhibited mitochondrial dysfunction, H_2_O_2_ production, and glutathione depletion mediated by AA + iron in the same cells. Meanwhile, the cytoprotective effects of BFE against oxidative stress were associated with the activation of AMP-activated protein kinase (AMPK). In particular, based on the histopathological observations, BFE (30 and 100 mg/kg) showed clear hepatoprotective effects against CCl_4_-induced acute hepatic damage. Furthermore, it inhibited 4-hydroxynonenal and nitrotyrosine immunoreactivity in hepatocytes. These results provide evidence that BFE has beneficial hepatoprotective effects against hepatic damage via the activation of AMPK pathway. Accordingly, BFE may have therapeutic potential for diverse liver disorders.

## 1. Introduction

The flower buds of* Buddleja officinalis* Maxim. are used as a folk remedy in traditional Oriental medicine.* B. officinalis* is a flowering shrub in the family Scrophulariaceae that is widely distributed in America, Africa, and Asia. It is used in China and Korea to treat inflammation, vascular diseases, conjunctivitis, headache, and stroke, as well as enhance liver function [1–3].* B. officinalis* reportedly contains iridoids (e.g., methylcatalpol and 6-O-vanilloyl ajugol), monoterpenoids (e.g., betulalbusides A), flavonoids (e.g., apigenin, isorhoifolin, and linarin), triterpenoids (e.g., acteoside, salidroside, and echinacoside), and phenylethanoids (e.g., buddlejasaponin I and mimengoside B) [1, 3–7].

Oxidative stress induces cell damage and is a major driver of the progression of many human disorders [8, 9]. High levels of reactive oxygen species (ROS) can alter membrane phospholipids [10], while fatty acid oxidation can damage cell signaling. In particular, arachidonic acid (AA), an omega-6 fatty acid, is involved in inflammation and contributes to the induction of necrosis and apoptosis [9, 11]. Moreover, AA and iron (AA + iron) synergistically produce more ROS and cause mitochondrial dysfunction and cell death [12, 13].

AMPK, a cellular energy gauge, is a major target for the treatment of metabolic disorders that has central roles in nutrient metabolism, energy homeostasis, cell survival, and apoptosis [12, 14]. Shin and Kim [12] showed that dithiolethiones protect hepatocytes from mitochondrial dysfunction and ROS production mediated by AA + iron via AMPK activation. Moreover, Dong et al. [15] suggested that red ginseng extract protects hepatocytes against AA + iron-induced oxidative stress through AMPK activation.

Several studies have observed the anti-inflammatory activity of* B. officinalis* flower extract (BFE), including downregulation of extracellular signal-regulated kinase (ERK) 1/2 and nuclear factor- (NF-) *κ*B signaling in BV-2 microglial cells, reduction of intracellular ROS production and NF-*κ*B in human umbilical vein endothelial cells (HUVECs), and inhibition of high glucose-induced matrix metalloproteinase (MMP) activity through the inhibition of oxidative stress in HUVECs [2, 16, 17]. However, no studies have determined whether BFE can protect hepatocytes from oxidative stress, and the properties of BFE on AMPK have not been evaluated. Therefore, we examined whether BFE protects hepatocytes from severe oxidative stress induced by AA + iron by inhibiting glutathione (GSH) depletion, hydrogen peroxide (H_2_O_2_) production, and mitochondrial dysfunction and whether the cytoprotective effects result from AMPK activation. Histomorphometric and histopathological analyses were performed to investigate the possible hepatoprotective effects of BFE against carbon tetrachloride- (CCl_4_-) induced hepatic damage in mice. The results revealed a molecular basis for the effects of BFE on hepatocyte protection.

## 2. Materials and Methods

### 2.1. Materials

AA and compound C were obtained from Calbiochem (San Diego, CA, USA). Anti-poly [ADP-ribose] polymerase (PARP), anti-procaspase-3, anti-phospho-AMPK*α*, and horseradish peroxidase- (HRP-) conjugated goat anti-mouse antibodies were obtained from Cell Signaling Technology (Beverly, MA, USA). HRP-conjugated goat anti-rabbit antibody was purchased from Thermo Fisher Scientific (Rockford, IL, USA). Dimethyl sulfoxide (DMSO) was obtained from Junsei Chemical (Tokyo, Japan). Anti-4-hydroxynonenal (HNE) polyclonal antibody was from Abcam (Cambridge, UK) and anti-nitrotyrosine (NT) polyclonal antibody was obtained from Millipore (Temecula, CA, USA). The VECTASTAIN Elite ABC HRP Kit and Peroxidase Substrate Kit were supplied from Vector Labs (Burlingame, CA, USA). Nitrilotriacetic acid, ferric nitrate, 3-(4,5-dimethylthiazol-2-yl)-2,5-diphenyltetrazolium bromide (MTT), 2′7′-difluorescin diacetate (DCFH-DA), rhodamine (Rh)123, anti-*β*-actin antibody, acacetin, apigenin, luteolin, and other chemicals were obtained from Sigma-Aldrich (St. Louis, MO, USA).

### 2.2. BFE Preparation

*B. officinalis* flowers were supplied from Daewon Pharmacy (Daegu, Republic of Korea), and a voucher specimen (DHU–GHF M87) was deposited at the College of Korean Medicine, Daegu Haany University, Korea. The BFE was prepared by extracting 100 g of* B. officinalis* flowers in 1.3 L of boiled water for 4 h. The BFE was filtered through a 0.22 *μ*m filter (Nalgene, New York, NY, USA), lyophilized with a vacuum evaporator, and stored at −20°C. The yield of lyophilized BFE was 12.5%. Dried BFE powder was dissolved in distilled water before use.

### 2.3. Chemical Profiling of BFE by Ultra Performance Liquid Chromatography (UPLC)

#### 2.3.1. Chromatography Conditions

For the separation, we used a UPLC system with a pump and ACQUITY UPLC (Waters, Milford, MA, USA), along with an ACQUITY photodiode array detector (Waters). We used the Empower Data System (Waters) to record the detector output. A Waters ACQUITY BEH C_18_ column (1.7 *μ*m, 2.1 mm × 100 mm) was used for the analysis. The column temperature was maintained at 25°C. The mobile phase was composed of acetonitrile with 0.1% formic acid and water, and the mixture was injected with sample (2 *μ*L) at a fluid velocity of 0.4 mL/min. For the photodiode array detector analysis, acacetin and luteolin were analyzed at 330 nm and apigenin was analyzed at 345 nm.

#### 2.3.2. Sample and Standard Solution Preparation

BFE was dissolved in methanol (10 mg/mL). Before the UPLC analysis, the sample was filtered through a 0.22 *μ*m filter. In addition, standard solutions of acacetin, apigenin, and luteolin were dissolved in methanol at a concentration of 1 *μ*g/mL and diluted to 1, 5, 10, and 20 ng/mL. The solutions were stored at 4°C.

### 2.4. Cell Culturing

HepG2 cells (American Type Culture Collection) were cultured in Dulbecco's modified Eagle's medium containing 50 mg/mL of streptomycin, 50 units/mL of penicillin, and 10% fetal bovine serum (FBS) at 37°C in a 5% carbon dioxide atmosphere. Before experimentation, the cells were cultured in medium without FBS (12 h) and then induced with 10 *μ*M of AA (12 h), followed by 5 *μ*M of iron (1 h). To determine the effects of BFE, the cells were pretreated with 10, 30, and 100 *μ*g/mL of BFE for 1 h before the AA treatment.

### 2.5. MTT Assay for Cell Viability

The MTT assay was performed as described previously [18]. Briefly, HepG2 cells were incubated in 24-well plates (5 × 10^4^ cells/well). The cells were stained with MTT (0.25 mg/mL, 2 h) after treatment with AA + iron or BFE (10, 30, and 100 *μ*g/mL) and AA + iron. The medium was removed from each well and 200 *μ*L of DMSO was added to dissolve the produced formazan crystals. Subsequently, absorbance was measured at 570 nm using a Titertek Multiskan automatic microplate reader (Tecan, Huntsville, AL, USA). Cell viability was defined relative to the control cells [viability (% of control) = (absorbance of treated sample)/(absorbance of control) × 100].

### 2.6. Preparation of Whole Cell Lysates and Immunoblot Analysis

Preparation of whole cell lysates and immunoblot analysis were conducted as described previously [18]. Briefly, cells were lysed in radioimmunoprecipitation assay (RIPA) buffer containing Halt Protease and Phosphatase Inhibitor Cocktail (Thermo Fisher Scientific) for 10 min and then centrifuged at 15,000 ×g for 30 min. The resulting supernatant was used as whole cell lysate. Equal amounts of protein were loaded onto sodium dodecyl sulfate-polyacrylamide gel electrophoresis (SDS-PAGE) gels and electrophoretically transferred onto a nitrocellulose membrane. The nitrocellulose membrane was incubated with the indicated primary antibody and then incubated with HRP-conjugated secondary antibody. The protein bands of interest were visualized using enhanced chemiluminescence (Amersham Biosciences, Buckinghamshire, UK). Equal protein loading was verified by *β*-actin immunoblotting. A densitometric analysis was conducted using ImageJ software (NIH, Bethesda, MD, USA).

### 2.7. Measurement of H_2_O_2_ Production

The amount of H_2_O_2_ was determined from the fluorescence intensity of cells treated with DCFH-DA (10 *μ*M) for 1 h at 37°C [15]. The fluorescence intensity was recorded at 485/530 nm (excitation/emission) using a Titertek Multiskan automatic microplate reader (Tecan).

### 2.8. Determination of Reduced GSH Content

The GSH concentration in HepG2 cells was measured using a GSH-400 kit (Oxis International, Portland, OR, USA) [12]. After treatment, cells were lysed with metaphosphoric acid and the reduced GSH concentration was measured with a spectrometer (Tecan, Research Triangle Park, NC, USA).

### 2.9. Measurement of MMP

MMP was measured via fluorescence-activated cell sorting (FACS; Partec, Görlitz, Germany) after staining cells with Rh123, a membrane-permeable fluorescent dye [12]. The treated cells were stained with 0.05 *μ*g/mL of Rh123 for 30 min and harvested via trypsinization. During the analysis, 20,000 events were recorded.

### 2.10. CCl_4_-Induced Liver Injury

Male ICR mice (6 weeks old, 30–33 g) were purchased from Orient Bio Inc. (Seongnam, Korea) and acclimatized for 1 week. To induce liver injury, CCl_4_ (0.5 mL/kg/day) dissolved in corn oil was intraperitoneally (i.p.) injected into the mice (*n* = 5/group) for 2 days as described by Zhao et al. with minor modification [19]. In addition, BFE dissolved in water was administered orally (p.o.) to the mice at doses of 30 or 100 mg/kg/day for 4 consecutive days. On day 4, the mice were injected with CCl_4_ 1 h after BFE treatment. All mice were sacrificed 24 h after the second CCl_4_ injection, and liver and blood samples were collected. All animal procedures were conducted in accordance with the national regulations regarding the usage and welfare of laboratory animals and were approved by the Institutional Animal Care and Use Committee of Daegu Haany University (Approval number: DHU2014-078).

### 2.11. Blood Biochemistry

Plasma alanine aminotransferase (ALT) and aspartate aminotransferase (AST) were measured by using analysis kits (Pointe Scientific Inc., Canton, MI, USA) and an automated blood chemistry analyzer (Photometer 5010; Robert Riele GmbH & Co. KG, Berlin, Germany) according to manufacturer's instructions [20].

### 2.12. Histological Process

Samples from the left lobes of the liver were separated and fixed in 10% formalin, embedded into paraffin, sectioned (thickness, 3-4 *μ*m), and stained with hematoxylin and eosin [21]. The histopathological profiles of the samples were examined under a microscope (Nikon, Tokyo, Japan). To identify more detailed changes, the percentage of degenerative regions (%/mm^2^) in the liver showing centrilobular necrosis, congestion, and inflammatory cell infiltration on hepatic lobules was calculated with an image analyzer (*i*Solution FL ver. 9.1; IMT* i*Solution Inc., Vancouver, Quebec, Canada). Furthermore, the number of hepatocytes exhibiting degenerative changes (i.e., acute cellular swelling and necrosis) and infiltrated inflammatory cells was recorded with an image analyzer as cells/1,000 hepatocytes and cells/mm^2^ of hepatic parenchyma [22] by a histologist blinded to the treatment groups.

### 2.13. Immunohistochemistry

Changes in the number of cells immunoreactive for 4-HNE and NT were assessed using primary antibodies with a peroxidase substrate and ABC kit (Vector Labs). Briefly, endogenous peroxidase activity was obstructed by incubation in 0.3% H_2_O_2_ and methanol for 0.5 h, and nonspecific binding of immunoglobulin was blocked with normal horse serum blocking solution for 1 h in a chamber after heating at 98–100°C following epitope retrieval in a 10 mM citrate buffer (pH 6.0) [21, 22]. Primary antisera were treated overnight in a chamber at 4°C and then incubated with a biotinylated secondary antibody and ABC reagent in a chamber at 25°C for 1 h. Finally, sections were reacted with the peroxidase substrate kit at 25°C for 3 min. All sections were washed three times in 0.01 M phosphate buffered saline. Positive cells were defined as having >20% 4-NHE and NT immunoreactivity. The number of immunolabeled cells located in a restricted field of view was calculated in the hepatic parenchyma surrounding central veins and centrilobular regions using an image analyzer by a histologist blinded to the treatment groups [22], denoted as cells/1,000 hepatocytes.

### 2.14. Statistical Analysis

We used one-way analysis of variance (ANOVA) to identify significant differences among the treatment groups. For each significant treatment effect, we used the Newman-Keuls test to compare multiple group means. Moreover, histomorphometric values were expressed as the means ± standard deviation of seven hepatic histological fields. A multiple comparison test of the different dose groups was performed. If Levene's test indicated no significant deviations from variance homogeneity, the data were analyzed with a one-way ANOVA followed by a least-significant differences multicomparison test to determine which pairs differed significantly. If significant deviations from variance homogeneity were detected by Levene's test, the nonparametric Kruskal-Wallis H (KWH) test was performed. When a significant difference was identified by the KWH test, a Mann–Whitney *U* test was conducted to determine which pairs differed significantly. Statistical analyses were performed in SPSS software (ver. 14.0K; SPSS Inc., Chicago, IL, USA). Results were considered to differ significantly when *p* < 0.05. In addition, the percentage point (pp) changes between the intact control and the CCl_4_ or 100 mg/kg BFE control were calculated to monitor the severity of hepatic damage induced in this study. The pp changes between the CCl_4_ and CCl_4_ + BFE 30 or 100 mg/kg treated livers were calculated to provide an understanding of their efficacy, as follows: pp change compared with the intact control (%) = ((data of CCl_4_ or BFE 100 mg/kg control − data of intact control)/data of intact control) × 100; pp change compared with the CCl_4_ (%) = ((data of CCl_4_ + BFE-treated mice − data of CCl_4_)/data of CCl_4_) × 100.

## 3. Results

### 3.1. Analysis of BFE

**Figure 1:**
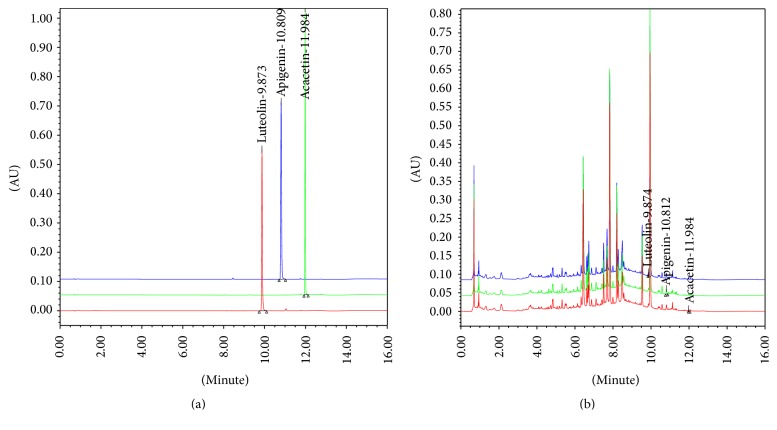
Ultra performance liquid chromatography (UPLC) chromatogram of the three major compounds identified in* B. officinalis* flower extract (BFE). (a) UPLC chromatogram of the commercial standard compounds. (b) UPLC chromatogram of the three major compounds in BFE. The chromatograms were obtained at 330 nm (acacetin and luteolin) and 345 nm (apigenin).

BFE was analyzed for its acacetin, apigenin, and luteolin content using UPLC. The concentrations of the three compounds were calculated from a calibration curve of standards (Table 1 and Figure 1). The method validation confirmed its stability and reliability and resulted in consecutive separation of the three major compounds in BFE.

### 3.2. Inhibition of AA + Iron-Induced Cell Death by BFE

**Figure 2:**
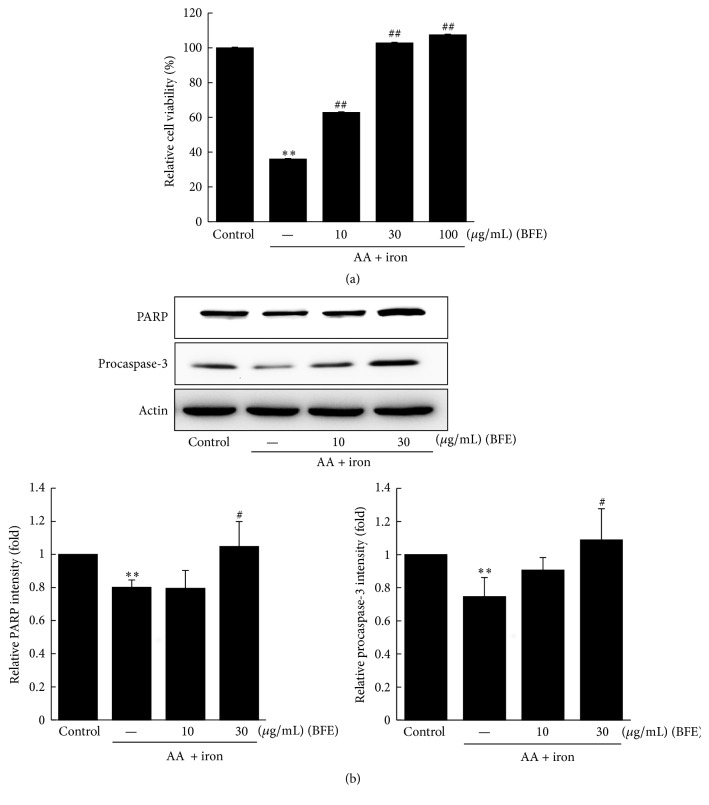
Inhibition of arachidonic acid (AA) + iron-induced cell death by BFE. (a) HepG2 cells were pretreated with 10, 30, and 100 *μ*g/mL of BFE for 1 h and subsequently incubated with 10 *μ*M of AA for 12 h, followed by exposure to 5 *μ*M of iron for 1 h. Cell viability was determined with the MTT assay. (b) Immunoblot analyses of proteins related to apoptosis were performed using HepG2 cell lysates incubated with 10 or 30 *μ*g/mL of BFE for 1 h, continuously treated with 10 *μ*M of AA for 12 h, and then exposed to 5 *μ*M of iron for 1 h. Equal protein loading was verified with *β*-actin immunoblotting. Data represent the means ± SD of three independent experiments relative to the control (^*∗∗*^*p* < 0.01 between the control and AA + iron-treated cells; ^#^*p* < 0.05 and ^##^*p* < 0.01 between the AA + iron-treated cells without or with BFE).

We examined the protective effects of BFE against AA + iron-induced hepatotoxicity using the MTT assay (Figure 2(a)). Treatment with AA + iron resulted in significantly decreased cell viability of HepG2 cells compared to the control (*p* < 0.01). However, further treatment with BFE (10, 30, and 100 *μ*g/mL) resulted in significantly increased cell viability (*p* < 0.01) (Lanes 3–5, Figure 2(a)). Furthermore, PARP and procaspase-3 protein levels were analyzed by Western blotting to evaluate the protective effects of BFE on AA + iron-induced cytotoxicity. Treatment with AA + iron significantly reduced levels of PARP and procaspase-3 (*p* < 0.01); however, the negative effects were significantly restored by BFE treatment (30 *μ*g/mL) (Figure 2(b)).

### 3.3. Effects of BFE on AA + Iron-Induced Oxidative Stress and Mitochondrial Dysfunction

**Figure 3:**
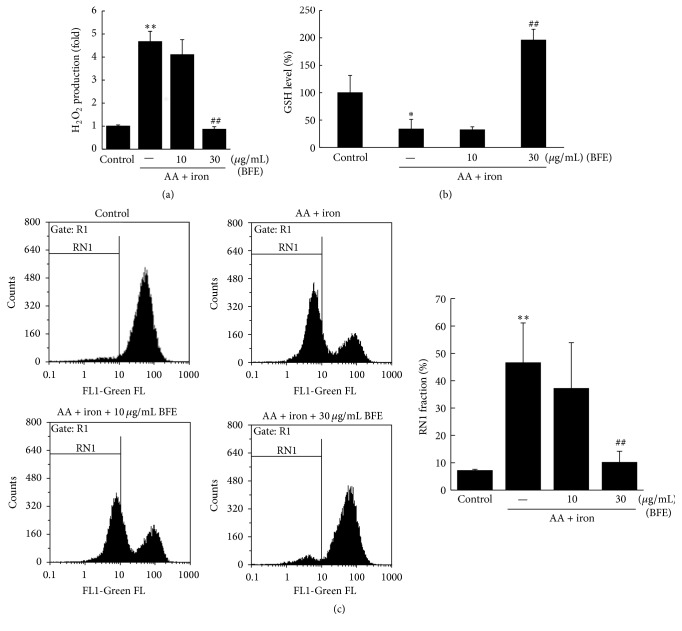
Effects of BFE on AA + iron-induced oxidative stress and mitochondrial dysfunction. HepG2 cells were treated with 10 or 30 *μ*g/mL of BFE for 1 h, continuously treated with 10 *μ*M of AA for 12 h, and then exposed to 5 *μ*M of iron for 1 h. (a) The amount of H_2_O_2_ was determined from the fluorescence intensity of cells treated with DCFH-DA (10 *μ*M) for 1 h at 37°C. (b) Analysis of glutathione (GSH) content. (c) After the cells were stained with 0.05 *μ*g/mL Rh123 for 30 min, the fluorescence intensity was measured with fluorescence-activated cell sorting (FACS) and the RN1 fraction (Rh123-negative cells) was represented as a percentage of the total. All data represent means ± SD of three independent experiments (^*∗*^*p* < 0.05 and ^*∗∗*^*p* < 0.01 between control and AA + iron-treated cells; ^##^*p* < 0.01 between AA + iron-treated cells without or with BFE).

We evaluated whether BFE inhibited H_2_O_2_ production in AA + iron-treated hepatocytes. Treatment with AA + iron significantly increased H_2_O_2_ levels (Figure 3(a)). However, BFE (30 *μ*g/mL) significantly inhibited the H_2_O_2_ produced by AA + iron exposure (*p* < 0.01). To verify the antioxidative effect of BFE, GSH levels were measured following a colorimetric method. Treatment with AA + iron significantly reduced the intracellular GSH concentrations in HepG2 cells (*p* < 0.05, Figure 3(b)), but pretreatment with BFE (30 *μ*g/mL) significantly inhibited this reduction (*p* < 0.01). Furthermore, the effects of AA + iron on mitochondrial dysfunction and protection through BFE were evaluated in HepG2 cells stained with Rh123. Treatment with AA + iron significantly increased the population of Rh123-negative cells (RN1 fraction) (*p* < 0.01, Figure 3(c)). In addition, treatment with BFE (30 *μ*g/mL) significantly inhibited the AA + iron-mediated increase in the RN1 fraction (*p* < 0.01, Figure 3(c)).

### 3.4. Effects of BFE on AMPK Activation

**Figure 4:**
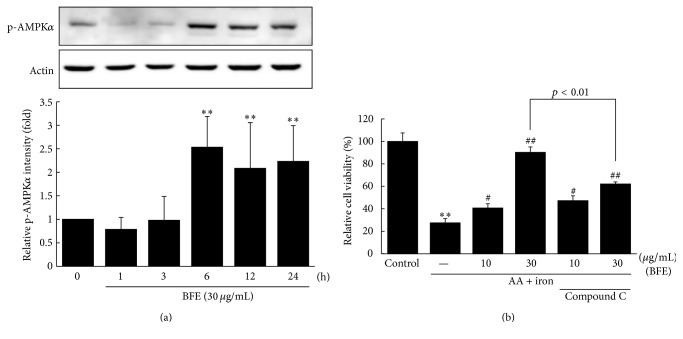
Effects of BFE on AMPK activation. (a) AMPK activation in cell lysate treated with 30 *μ*g/mL of BFE for the indicated time periods. Equal protein loading was verified by *β*-actin immunoblotting. A representative blot from three independent experiments is shown. Relative protein levels of activated AMPK were analyzed by densitometry. (b) The role of AMPK on BFE-mediated cytoprotection. Cell viability was performed on HepG2 cells incubated without or with 10 *μ*M of compound C for 1 h and continuously treated with BFE (10 or 30 *μ*g/mL, 6 h) and AA + iron, as described in Figure 2(a). Data represent means ± SD of three independent experiments [(a) ^*∗∗*^*p* < 0.01 between the control and BFE-treated cells; (b) ^*∗∗*^*p* < 0.01 between control and AA + iron-treated cells; ^#^*p* < 0.05 and ^##^*p* < 0.01 between AA + iron-treated cells without or with BFE or compound C].

We investigated the time-course effects of BFE on AMPK*α* phosphorylation. BFE (30 *μ*g/mL) resulted in significant increases in AMPK*α* phosphorylation in HepG2 cells after 6, 12, and 24 h (Figure 4(a)). In addition, the AMPK-inhibitory effects on cell viability were evaluated using compound C. Treatment with the AMPK inhibitor compound C significantly inhibited the cell viability induced by BFE (30 *μ*g/mL) in the presence of AA + iron, indicative of the inhibition of AMPK activation (*p* < 0.01, Figure 4(b)).

### 3.5. Hepatoprotective Effects of BFE on CCl_4_-Induced Liver Injury

**Figure 5:**
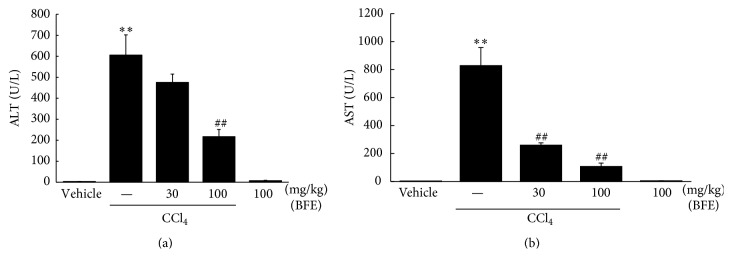
Effects of BFE on CCl_4_-induced liver injury. (a and b) Plasma alanine aminotransferase (ALT) and aspartate aminotransferase (AST) activities. ICR mice (*n* = 5/group) were treated with BFE (30 or 100 mg/kg/day, p.o., for 4 days). On day 4, the mice were injected with CCl_4_ (0.5 mL/kg/day, i.p.) 1 h after treatment with BFE. Data represent means ± SD of five mice (^*∗∗*^*p* < 0.01 compared to the vehicle-treated group; ^##^*p* < 0.01 compared to the CCl_4_-treated group).

We evaluated the effects of CCl_4_ on plasma ALT and AST levels. Treatment with CCl_4_ caused hepatocyte toxicity, represented by significant increases in plasma ALT and AST activities (Figures 5(a) and 5(b)). When we investigated the effects of treatment with 30 or 100 mg/kg of BFE on CCl_4_-induced liver injury, BFE (100 mg/kg) reduced liver injury in mice, showing significant decreases in ALT and AST activities (^##^*p* < 0.01, Figures 5(a) and 5(b)). These findings indicated that 100 mg/kg of BFE protected the liver against CCl_4_-induced toxicity.

### 3.6. Histological Examination

**Figure 6:**
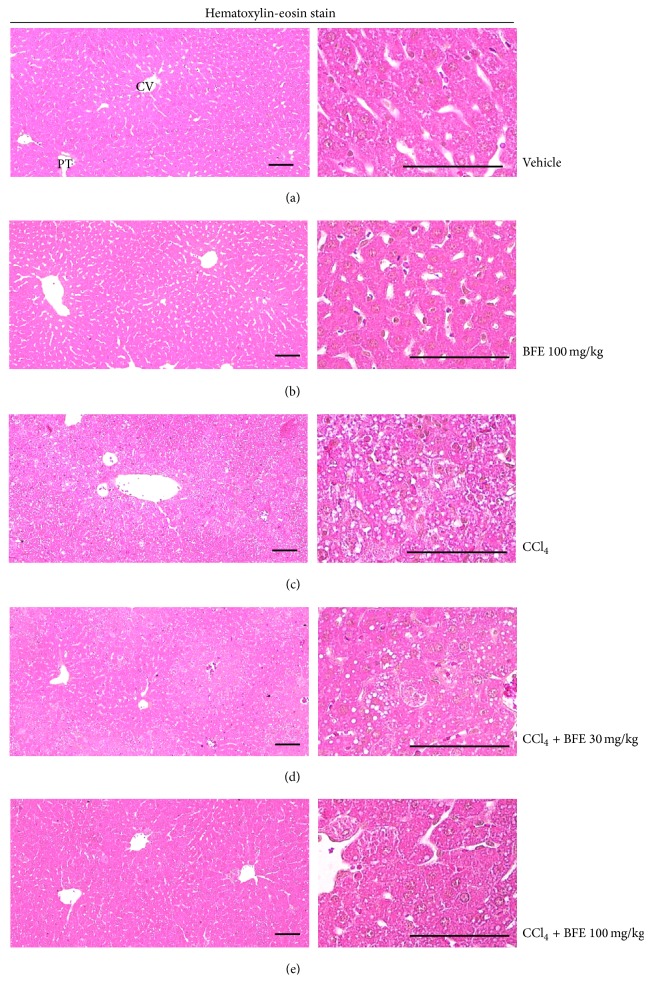
Representative histological profiles of hepatic tissue collected from intact or acute CCl_4_-treated mice with or without BFE coadministration. Vacuolation and degenerative changes (i.e., ballooning, fatty changes, and inflammatory cell infiltration) were detected in the CCl_4_-treated mice; however, this CCl_4_ treatment-related acute hepatic damage was significantly inhibited by treatment with 30 and 100 mg/kg of BFE in a dose dependent manner. No meaningful histopathological changes were observed in the 100 mg/kg BFE-treated control mice compared with the intact control mice. (a) Vehicle-treated intact control mice. (b) 100 mg/kg BFE-treated mice. (c) CCl_4_-treated mice. (d) CCl_4_ + 30 mg/kg BFE-treated mice (lower dosage test group). (e) CCl_4_ + 100 mg/kg BFE-treated mice (higher dosage test group). CV: central vein; PT: portal triad. Scale bars: 120 *μ*m.

**Figure 7:**
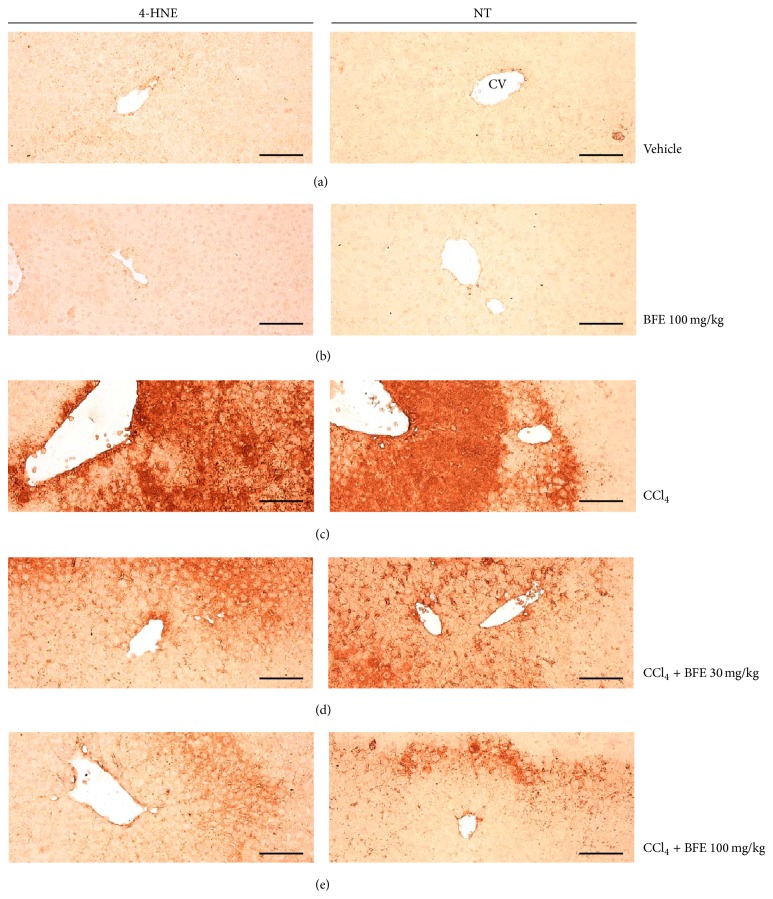
Representative 4-HNE and NT-immunolabeled cells in hepatic tissue collected from intact or acute CCl_4_-treated mice with or without BFE coadministration. Noticeable increases in 4-HNE- and NT-positive cells (deep brown colored cells) were observed in the CCl_4_ control mice compared with the intact control mice, but they were significantly reduced by treatment with 30 and 100 mg/kg of BFE, indicative that dosages of BFE over 30 mg/kg inhibited lipid peroxidation [i.e., formation of 4-HNE] and inducible nitric oxide synthase- (iNOS-) related oxidative stress [i.e., NT immunoreactivity], at least in the model used in this study. No meaningful changes in the 4-HNE and NT immunoreactivities were observed in the 100 mg/kg BFE-treated control mice compared with the intact control mice. (a) Vehicle-treated intact control mice. (b) 100 mg/kg BFE-treated mice. (c) CCl_4_-treated mice. (d) CCl_4_ + 30 mg/kg BFE-treated mice (lower dosage test group). (e) CCl_4_ + 100 mg/kg BFE-treated mice (higher dosage test group). 4-HNE: 4-hydroxynonenal, NT: nitrotyrosine, CV: central vein, and scale bars: 120 *μ*m.

The results of the histomorphometric analysis are listed in Tables 2 and 3, and representative histological profiles of hepatic tissue with representative 4-HNE and NT-immunolabeled cells are shown in Figures 6 and 7, respectively. As CCl_4_ intoxication progressed, degenerative changes in the liver were detected under histopathological observation of the lateral lobes, representative of centrilobular necrosis, and observed as ballooning of hepatocytes, deposition of lipid droplets in hepatocytes (cells with fatty changes), and infiltration of inflammatory cells. The CCl_4_-induced acute hepatic damage was confirmed with histomorphometry as the percentage of degenerative regions, number of degenerative hepatocytes, and number of inflammatory cells infiltrated in the hepatic parenchyma or hepatocytes, which were significantly (*p* < 0.01) higher in the CCl_4_-treated cells compared with the intact control. However, the CCl_4_-induced acute hepatic damage was significantly (*p* < 0.01) inhibited by treatment with 30 and 100 mg/kg of BFE in a dose dependent manner. No meaningful histopathological changes were observed in the 100 mg/kg BFE-treated control mice compared with the intact control mice. The percentage of hepatic degenerative regions in the 100 mg/kg BFE and CCl_4_ groups changed by −3.09 and 3,461 pp compared with the intact control, while they changed by −26.86 and −76.21 pp in the CCl_4_ + BFE- (30 and 100 mg/kg) treated mouse livers compared with the CCl_4_ group, respectively. Moreover, the number of degenerative hepatocytes in the 100 mg/kg BFE and CCl_4_ groups changed by −1.08 and 5,979.57 pp compared with the intact control but changed by −31.68 and −77.75 pp in the CCl_4_ + BFE- (30 and 100 mg/kg) treated mouse livers compared with the CCl_4_ group, respectively. In particular, the number of infiltrated inflammatory cells in the 100 mg/kg BFE and CCl_4_ groups changed by −2.90 and 1,982.61 pp compared with the intact control but changed by −44.40 and −74.81 pp in CCl_4_ + BFE- (30 and 100 mg/kg) treated mouse livers compared with the CCl_4_ group, respectively (Table 2 and Figure 6).

Notably, significant (*p* < 0.01) increases of 4-HNE and NT-immunolabeled cells were observed in the CCl_4_ group compared with the intact control, respectively. However, the lower (30 mg/kg) and higher (100 mg/kg) BFE dosages significantly (*p* < 0.01) reduced 4-HNE and NT immunoreactivities compared with CCl_4_ alone. No meaningful histopathological changes were demonstrated in the 100 mg/kg BFE-treated control mice compared with the intact control mice. The number of 4-HNE-positive cells in the 100 mg/kg BFE and CCl_4_ groups changed by −2.44 and 5629.27 pp compared with the intact control, while they changed by −42.23 and −72.90 pp in the CCl_4_ + BFE- (30 and 100 mg/kg) treated mouse livers compared with the CCl_4_ group, respectively. In addition, the number of NT-positive cells in the 100 mg/kg BFE and CCl_4_ groups changed by −1.69 and 7,516.95 pp compared with the intact control, while they changed by −62.91 and −88.94 pp in the CCl_4_ + BFE- (30 and 100 mg/kg) treated mouse livers compared with the CCl_4_ group, respectively (Table 3 and Figure 7).

### 3.7. Effects of BFE and Its Compounds on AA + Iron-Induced Cell Death, AMPK Inactivation, and Mitochondrial Dysfunction

**Figure 8:**
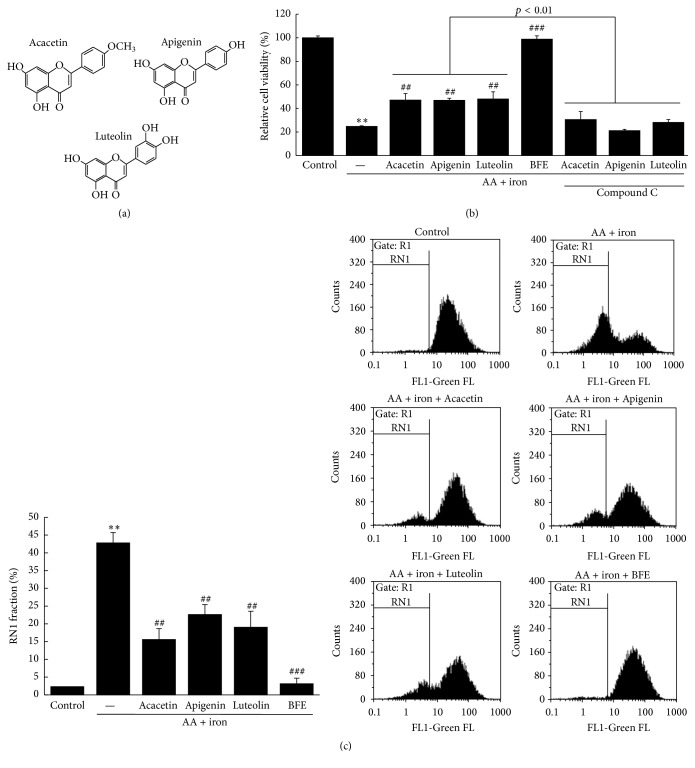
Effects of BFE and its compounds on AA + iron-induced cell death, AMPK inactivation, and mitochondrial dysfunction. (a) Chemical structures of the BFE compounds. (b) The effects of BFE and its compounds on cell viability and AMPK activation. The cytoprotective effects of BFE (30 *μ*g/mL) and its compounds (10 *μ*M each) against AA + iron with or without compound C (10 *μ*M) were evaluated using the MTT assay. (c) The effects of BFE and its compounds on mitochondrial dysfunction. After staining the cells with 0.05 *μ*g/mL of Rh123 for 30 min, the fluorescence intensity was measured with FACS and RN1 fractions are represented as a percentage of the total. All data represent means ± SD of three independent experiments [(b) ^*∗∗*^*p* < 0.01 between the control and AA + iron-treated cells; ^##^*p* < 0.01 and ^###^*p* < 0.001 between the AA + iron-treated cells without or with BFE or its compounds with or without compound C; (c) ^*∗∗*^*p* < 0.01 between control and AA + iron-treated cells; ^##^*p* < 0.01 and ^###^*p* < 0.001 between AA + iron-treated cells without or with BFE and its compounds].

We investigated the protective effects of BFE and its compounds (acacetin, apigenin, and luteolin) against the hepatotoxic effects of AA + iron using the MTT assay (Figure 8(a)). In addition, the AMPK-inhibitory effects on cell viability using compound C were examined to confirm AMPK activation in cells treated with the BFE compounds. Treatment with AA + iron resulted in significantly decreased cell viability compared to the control (*p* < 0.01). However, treatment with BFE compounds (10 *μ*M) significantly increased cell viability compared to the AA + iron-treated group (*p* < 0.01), but not in the groups treated with compound C (Figure 8(b)). These findings suggest that the BFE compounds activate the AMPK pathway. The effects of AA + iron on mitochondrial dysfunction and the protective effects of the BFE compounds were evaluated in HepG2 cells stained with Rh123. Treatment with AA + iron significantly increased the RN1 fraction (*p* < 0.01, Figure 8(c)). In addition, treatment with BFE compounds (10 *μ*M) significantly inhibited the AA + iron-mediated increase in the RN1 fraction (*p* < 0.01, Figure 8(c)).

## 4. Discussion

In traditional Oriental medicine, BFE is used as a folk remedy to treat inflammation, vascular diseases, conjunctivitis, clustered nebulae, headache, and stroke, as well as enhance liver function [1–3]. However, there is little scientific data to support the effects of BFE. Accordingly, we investigated the benefits of BFE on the protection of hepatocytes in vivo and in vitro.

Oxidative stress is linked to many human diseases via the cellular dysfunction induced by excessive ROS production [8–10]. For example, membrane phospholipids can be altered by high ROS levels [10]. AA released from membranes increases the inflammatory response, apoptosis, and oxidative stress [9, 12, 23]. Moreover, excessive iron increases the AA-releasing capacity by changing membrane phospholipids [24, 25], while AA synergizes the capability of iron to elevate mitochondrial damage and oxidative stress, thereby evoking toxicity in hepatocytes [12, 23]. In this study, AA + iron-treated hepatocytes were evaluated as an in vitro model to examine the antioxidative effects of BFE. A previous study found that isoliquiritigenin inhibited AA + iron-stimulated apoptosis by modifying PARP and procaspase-3 cleavage [26]. Procaspases are inactive precursors of caspases, which require cleavage to produce the active enzyme and caspase-3 is responsible for the cleavage of important nuclear targets in the apoptotic pathway [27–29]. Our results revealed that treatment with AA + iron significantly decreased cell viability, but at the concentrations tested (10, 30, and 100 *μ*g/mL), treatment with BFE significantly increased cell viability. In addition, treatment with AA + iron significantly reduced PARP and procaspase-3 protein levels, and the negative effects were significantly restored by BFE treatment. Taken together, these results suggest that BFE may protect hepatocytes against AA + iron-induced apoptosis. Therefore, BFE might have therapeutic capability for liver diseases.

In a previous study, AA + iron-mediated cellular H_2_O_2_ production and decreases in GSH were significantly attenuated by treatment with red ginseng extract [15]. Our results indicated that AA + iron significantly increased levels of H_2_O_2_, but treatment with BFE (30 *μ*g/mL) significantly inhibited the H_2_O_2_ production mediated by AA + iron. In addition, AA + iron significantly reduced GSH concentration in the HepG2 cells, but BFE (30 *μ*g/mL) significantly inhibited the reduction. These findings reveal that BFE inhibits AA + iron-induced oxidative stress. Moreover, treatment with AA + iron significantly increased the RN1 fraction; however, treatment with BFE (30 *μ*g/mL) significantly inhibited the AA + iron-mediated increase in the RN1 fraction. This is comparable to a study that found that isorhamnetin protected hepatocytes by inhibiting AA + iron-mediated mitochondrial dysfunction [18]. These findings suggest that BFE protects hepatocytes by inhibiting AA + iron-induced mitochondrial dysfunction.

AMPK has key roles in cell survival, energy homeostasis, and apoptosis in response to oxidative stress [12, 14]. The regulatory role is demonstrated by increases in cell viability by the AMPK activators, including 5-aminoimidazole-4-carboxamide-1-*β*-d-ribofuranoside [30]. Dithiolethiones protect hepatocytes from the ROS production and mitochondrial dysfunction induced by AA + iron via AMPK activation [12]. Our results showed that treatment with BFE significantly increased the phosphorylation of AMPK*α* in HepG2 cells after 6, 12, and 24 h compared with the control group. Furthermore, the AMPK inhibitor compound C significantly inhibited the cell viability mediated by BFE (30 *μ*g/mL) in the presence of AA + iron. These findings suggest that BFE activates the AMPK pathway and that this activation is positively related to cell survival against oxidative stress.

CCl_4_ is widely used to induce liver injury in animal models via its damaging effects on hepatocytes. This chemical changes mitochondria and plasma membrane permeability in the liver and forms toxic reactive free radicals presumably through the cytochrome p450 2E1 pathway. A single injection of CCl_4_ causes necrosis of parenchymal cells in the liver lobule (zone 3) and its repeated treatment over long periods causes chronic liver diseases, such as fibrosis and cirrhosis [31, 32]. ALT and AST, biochemical markers of damaged hepatocytes, are produced by injured liver cells and released into the blood [33]. ALT is found in large quantities in the hepatocytes cytoplasm and is a main marker of liver damage. In addition, AST is present in several body tissues and its activities are increased in the presence of necrosis of skeletal muscles and liver cells. In this study, CCl_4_ caused hepatocyte toxicity in mice, as shown by significant increases in ALT and AST activities. Moreover, BFE (100 mg/kg) attenuated mice liver injury, as indicated by significant decreases in ALT and AST levels. These results suggest that BFE protects the liver against CCl_4_-induced toxicity.

Similar to previous reports [21, 22, 34, 35], vacuolation (i.e., deposition of lipid droplets), ballooning of hepatocytes, and inflammatory cell infiltration were detected in all CCl_4_-treated mice in this study, representative of classic centrilobular necrosis [21, 22, 34, 36]. The damaged hepatocytes were mainly located around the central veins, while cells with fatty changes were marginally located. This CCl_4_-related acute hepatic damage was indexed under histomorphometric examination as the percentage of degenerative regions, number of degenerative hepatocytes, and number of inflammatory cells infiltrated in hepatic parenchyma or hepatocytes, which were significantly higher in the CCl_4_ group compared with the intact control. However, the CCl_4_ treatment-related acute hepatic damage was significantly inhibited by treatment with 30 and 100 mg/kg of BFE in a dose dependent manner. These findings provide evidence that dosages over 30 mg/kg of BFE have beneficial hepatoprotective effects against CCl_4_-induced acute hepatic damage, at least under the conditions of this study.

4-HNE is an *α*, *β*-unsaturated hydroxyalkenal produced by lipid peroxidation in cells and is considered as a possible causal agent of numerous diseases, including chronic inflammation, neurodegenerative diseases, adult respiratory distress syndrome, atherogenesis, diabetes, and different types of cancer [22, 37–39]. In addition, CCl_4_ metabolism initiates the peroxidation of polyunsaturated fatty acids, producing *α*, *β*-unsaturated aldehydes, including 4-HNE and malondialdehyde [40, 41]. In this study, significant increases in 4-HNE-positive cells were observed in the CCl_4_ group compared with the intact control, but they were significantly reduced by treatment with both the lower (30 mg/kg) and higher (100 mg/kg) dosages of BFE with evident dose dependencies, respectively, representing direct evidence that dosages over 30 mg/kg of BFE inhibit lipid peroxidation and the formation of 4-HNE and protect against the hepatocyte necrotic changes induced by CCl_4_, at least in the model used in this study. Moreover, oral treatment with 100 mg/kg of BFE did not influence hepatic 4-HNE immunoreactivity in the intact normal mice. NT is a product of tyrosine nitration mediated by reactive nitrogen species, such as the peroxynitrite anion and nitrogen dioxide. It is detected in many pathological conditions, including CCl_4_-induced acute hepatic damage, and is considered as a marker of nitric oxide-dependent and reactive nitrogen species-induced nitrative stress [22, 42–44]. In this study, significant increases in NT-immunoreactive cells were observed in the CCl_4_ group compared with the intact control, similar to the 4-HNE immunopositive cells; however, they were significantly reduced by treatment with 30 and 100 mg/kg of BFE in a dose dependent manner. These results are considered as additional evidence that dosages of BFE over 30 mg/kg inhibit iNOS-related oxidative stress and protect against necrotic changes in hepatocytes induced by CCl_4_, at least in the animal model used in this study. However, oral treatment with 100 mg/kg of BFE did not influence the hepatic NT immunoreactivity of the intact normal mice, similar to the 4-HNE immunoreactivity results.

BFE is an aqueous extract from the flowers of* B. officinalis* that contains numerous bioactive compounds, including acteoside, apigenin, buddlejasaponin I, echinacoside, isorhoifolin, linarin, methylcatalpol, mimengoside B, 6-O-vanilloyl ajugol, and salidroside [1, 3–7]. Our results of the chromatographic analysis revealed that the main compounds in BFE were acacetin, apigenin, and luteolin. Acacetin (5,7-dihydroxy-4′-methoxyflavone) is a flavone compound that has been reported to have anti-inflammatory, antiperoxidative, and anticancer effects [45–47]. In addition, flavonoid apigenin (4′,5,7-trihydroxyflavone) has remarkable antioxidant, anti-inflammatory, and anticarcinogenic activities [48–50]. This compound has been shown to increase intracellular GSH levels by transactivation of promoters against oxidative stress [51]. Finally, luteolin (3′,4′,5,7-tetrahydroxyflavone) is a flavonoid that exists in several medicinal herbs and has pharmacological effects, including antioxidant and antimicrobial activities [52]. Extracts of plants rich in luteolin are widely used as traditional remedies for inflammatory disorders, hypertension, and cancer [53]. The results of the protective effects of BFE and its compounds (acacetin, apigenin, and luteolin) against AA + iron-mediated hepatotoxicity indicated that AA + iron treatment significantly decreased cell viability, but BFE (30 *μ*g/mL) and its compounds (10 *μ*M each) significantly increased cell viability. Furthermore, the AMPK inhibitor compound C significantly inhibited the cell viability promoted by treatment with BFE compounds in the presence of AA + iron. These findings reveal that BFE compounds activate the AMPK pathway, which is responsible for the protection against oxidative stress. In addition, AA + iron treatment significantly increased the RN1 fraction. However, the BFE compounds (10 *μ*M each) significantly inhibited the AA + iron-mediated increase in the RN1 fraction. Our findings demonstrate that BFE compounds protect hepatocytes by inhibiting AA + iron-mediated mitochondrial dysfunction. In this study, the hepatoprotective effects of BFE on oxidative stress may have been due to the BFE compounds, acacetin, apigenin, and luteolin. Although the three compounds showed hepatoprotective effects and AMPK activation, these effects were lower than those of BFE. Therefore, the protective effects of the herbal extract against cytotoxicity may be better than those of the isolated compounds alone due to synergistic effects.

## 5. Conclusions

Our results showed that BFE inhibited the AA + iron-mediated hepatotoxicity of HepG2 cells. In particular, BFE inhibited mitochondrial dysfunction, H_2_O_2_ production, and GSH depletion mediated by AA + iron in the same cells. Meanwhile, the cytoprotective effects of BFE against oxidative stress were associated with AMPK activation. In particular, based on the histopathological observations, BFE (30 and 100 mg/kg) showed obvious hepatoprotective effects against CCl_4_-induced acute hepatic damage under histopathological inspection and inhibited the immunoreactivity of 4-HNE and NT in hepatocytes, under immunohistochemical examination. These results provide evidence that BFE has beneficial hepatoprotective effects against hepatic damage through the activation of AMPK pathway. Accordingly, BFE may have therapeutic potential for diverse liver disorders.

## Figures and Tables

**Table 1:** Contents of three marker compounds of BFE by UPLC.

Compound	Content (ppm)
Acacetin	6.02 ± 0.014
Luteolin	24.20 ± 0.015
Apigenin	19.04 ± 0.012

Values are expressed as means ± SD of three independent experiments. BFE was analyzed for its acacetin, apigenin, and luteolin content using UPLC. BFE: *Buddleja  officinalis *Maxim. flower extract. UPLC: ultra performance liquid chromatography.

**Table 2:** General histomorphometrical analysis of acute CCl_4_-treated mouse hepatic tissues.

Groups	Percentages of degenerative regions (%/mm^2^ of hepatic parenchyma)	Numbers of inflammatory cells infiltrated (cells/mm^2^ of hepatic parenchyma)	Numbers of degenerative hepatocytes (cells/1000 hepatocytes)
Controls			
Intact	1.62 ± 0.63	6.90 ± 3.60	9.30 ± 5.06
BFE (100 mg/kg)	1.57 ± 0.80	6.70 ± 3.43	9.20 ± 4.76
CCl_4_	57.69 ± 10.64^a^	143.70 ± 33.66^a^	565.40 ± 119.48^a^
CCl_4_ + BFE-treated mice			
30 mg/kg	42.20 ± 10.93^ab^	79.90 ± 20.46^ab^	386.30 ± 102.54^ab^
100 mg/kg	13.73 ± 8.85^ab^	36.20 ± 11.38^ab^	125.80 ± 55.11^ab^

Values are expressed as means ± SD of 10 histological fields. CCl_4_: carbon tetrachloride; BFE: *Buddleja officinalis *Maxim. flower extract.

^a^ *p* < 0.01 as compared with intact control by MW test; ^b^*p* < 0.01 as compared with CCl_4_ control by MW test.

**Table 3:** Numbers of 4-HNE- and NT-immunoreactive cells on the hepatic parenchyma in acute CCl_4_-treated mouse hepatic tissue.

Groups	4-HNE (cells/1,000 hepatocytes)	NT (cells/1,000 hepatocytes)
Controls		
Intact	8.20 ± 2.70	5.90 ± 1.97
BFE (100 mg/kg)	8.00 ± 3.16	5.80 ± 2.53
CCl_4_	469.80 ± 55.65^a^	449.40 ± 105.77^a^
CCl_4_ + BFE-treated mice		
30 mg/kg	271.40 ± 54.23^ab^	166.70 ± 40.43^ab^
100 mg/kg	127.30 ± 15.97^ab^	49.70 ± 12.19^ab^

Values are expressed as means ± SD of 10 histological fields. CCl_4_: carbon tetrachloride; BFE: *Buddleja*  *officinalis *Maxim. flower extract. ^a^*p* < 0.01 as compared with intact control by MW test; ^b^*p* < 0.01 as compared with CCl_4_ control by MW test.

## Abbreviations

AA: Arachidonic acid
ABC: Avidin-biotin-peroxidase complex
ALT: Alanine aminotransferase
AMPK: AMP-activated protein kinase
ANOVA: Analysis of variance
AST: Aspartate aminotransferase
BFE: * Buddleja officinalis* Maxim. flower extract
CCl_4_: Carbon tetrachloride
DCFH-DA: 2′,7′-Dichlorofluorescein diacetate
DMSO: Dimethyl sulfoxide
FACS: Fluorescence-activated cell sorter
FBS: Fetal bovine serum
GSH: Glutathione
4-HNE: 4-Hydroxynonenal
MMP: Mitochondrial membrane potential
MTT: 3-(4,5-Dimethylthiazol-2-yl)-2,5-diphenyl-tetrazolium bromide
NT: Nitrotyrosine
PARP: Poly(ADP-ribose)polymerase
Rh 123: Rhodamine 123
ROS: Reactive oxygen species
UPLC: Ultra performance liquid chromatography.
